# fMRI as a molecular imaging procedure for the functional reorganization of motor systems in chronic stroke

**DOI:** 10.3892/mmr.2013.1603

**Published:** 2013-07-26

**Authors:** ASIMINA LAZARIDOU, LOUKAS ASTRAKAS, DIONYSSIOS MINTZOPOULOS, AZADEH KHANCHICEH, ANEESH SINGHAL, MICHAEL MOSKOWITZ, BRUCE ROSEN, ARIA TZIKA

**Affiliations:** 1 NMR Surgical Laboratory, Massachusetts General Hospital and Shriners Burn Institute, Harvard Medical School, Boston, MA, USA; 2 Radiology, Athinoula A. Martinos Center for Biomedical Imaging, Boston, MA, USA; 3 Mechanical Engineering, Northeastern University, Boston, MA, USA; 4 Department of Neurology, Stroke Research Center, Massachusetts General Hospital, Harvard Medical School, Boston, MA, USA

**Keywords:** functional magnetic resonance imaging, stroke, brain, dynamical causal modeling

## Abstract

Previous brain imaging studies suggest that stroke alters functional connectivity in motor execution networks. Moreover, current understanding of brain plasticity has led to new approaches in stroke rehabilitation. Recent studies showed a significant role of effective coupling of neuronal activity in the SMA (supplementary motor area) and M1 (primary motor cortex) network for motor outcome in patients after stroke. After a subcortical stroke, functional magnetic resonance imaging (fMRI) during movement reveals cortical reorganization that is associated with the recovery of function. The aim of the present study was to explore connectivity alterations within the motor-related areas combining motor fMRI with a novel MR-compatible hand-induced robotic device (MR_CHIROD) training. Patients completed training at home and underwent serial MR evaluation at baseline and after 8 weeks of training. Training at home consisted of squeezing a gel exercise ball with the paretic hand at ~75% of maximum strength for 1 h/day, 3 days/week. The fMRI analysis revealed alterations in M1, SMA, PMC (premotor cortex) and Cer (cerebellum) in both stroke patients and healthy controls after the training. Findings of the present study suggest that enhancement of SMA activity could benefit M1 dysfunction in stroke survivors. These results also indicate that connectivity alterations between motor areas might assist the counterbalance of a functionally abnormal M1 in chronic stroke survivors and possibly other patients with motor dysfunction.

## Introduction

Stroke is the third leading cause of death and the leading cause of long-term disability in the US (1). Approximately 4 million Americans live with the negative consequences of stroke (2,3). In addition, the lives of caregivers including spouses, children and friends are personally affected because of this significant disease. Current investigations have focused on stroke rehabilitation and brain plasticity as a mechanism in recovery (4).

Therefore, plasticity following stroke remains a crucial issue for stroke survivors and there is invariably some degree of functional recovery (5). In other words, when neurons are damaged by stroke, other neurons take over for them. This adaptive behavior assists in the reorganization of the brain and recovery of lost skills. Brain plasticity is therefore the reason intensive therapy is such a critical component of stroke rehabilitation (6–8).

Plasticity after stroke has traditionally been studied by observing changes only in the spatial distribution and laterality of focal brain activation during affected limb movement (9). However, neural reorganization is multifaceted and our understanding may be enhanced by examining dynamics of activity within large-scale networks involved in the sensorimotor control of the limbs. In stroke rehabilitation, functional imaging studies of the motor system have described task-related brain activation in recovered patients over and above control subjects in contralesional sensorimotor and premotor cortex, ipsilesional cerebellum, bilateral supplementary motor area (SMA) and parietal cortex (10–14).

Functional magnetic resonance imaging (fMRI) as a molecular magnetic resonance imaging procedure is used in various studies for stroke plasticity. This technique is based on the fact that the magnetic properties of deoxygenated and oxygenated hemoglobin in the blood are different and produce different signals (contrast) when imaged with T2* sensitive MRI sequences (15,16). Therefore, the mapping of the brain’s networks often begins by identifying a set of links, and then attempts to estimate the set of connections between these nodes, based on an analysis of the fMRI time series associated with these nodes. In most cases, the directionality of these links exhibits ‘connectivity’ by demonstrating how information flows through the network (17). Functional connectivity is a promising means of assessing the consequences of a stroke lesion as well as studying plasticity in neural networks.

A large number of neurological impairments that involve muscle weakness, loss of range of motion, and impaired force generation create deficits in motor control that affect the stroke survivor’s capacity for independent living and economic self-sufficiency (18). Tactile sensibility of the hand is essential for identifying objects and for motor performance. This performance is largely affected by stroke as well as sensory perception, which is difficult to recover. Many traditional therapeutic interventions have been used in rehabilitation to promote functional recovery, with outcome studies yielding inconsistent results (19). Recent evidence has demonstrated that intensive massed and repeated practice may be necessary to modify neural organization and effect recovery of functional motor skills (9).

A recent preliminary study on 4 individuals post-stroke showed that all 4 individuals improved in sensory tasks and motor performance, effects that remained 4 weeks post-treatment (20). In terms of traditional therapy, which is provided in a rehabilitation center or hospital, the patient is usually seen for half-hour sessions, once or twice a day. This visitation is decreased to once or twice a week in outpatient therapy. It is evident that in this service-delivery model, it is difficult to provide the amount or intensity of practice needed to effect neural and functional changes. Therefore, further intervention is required, including exercise tasks throughout the therapy. More recently, clinical studies using robot-assisted therapy have been shown to benefit patients during neurological recovery (21–32). The incremental improvements in clinical scales following intensive robotic therapy, although small, are statistically significant and certainly meaningful to patients.

In a previous study (8), we demonstrated decreased intrinsic neural coupling between M1 and cerebellum (Ce), which was consistent with a dysfunctional M1 to Ce connection in stroke patients compared to controls. Stroke patients also showed increased SMA to M1 and SMA to cerebellum coupling, suggesting that changes in SMA and Ce connectivity may occur to compensate for a dysfunctional M1. In this study, we present additional findings exploring whether training effective connectivity strengths altered after training relative to baseline and promote functional recovery in chronic stroke patients and healthy controls.

## Materials and methods

### Participants

Twelve healthy volunteers and 5 chronic stroke patients provided written informed consent to participate in this cross-sectional study. All experiments were approved by the Institutional Review Board at Massachusetts General Hospital and performed at the Athinoula A. Martinos Center for Biomedical Imaging. All participants used an MR-compatible hand induced robotic device (MR_CHIROD) during fMRI at 45% of their maximum strength. The brain maps and connectivity strengths of stroke patients and healthy controls were compared prior and subsequent to the training.

### MR_CHIROD hand device

The design and testing of the hand device have been previously described (4,8,33). The hand device consists of three main subsystems: i) an electrorheological fluid (ERF) resistive element; ii) handles and iii) two sensors, an optical encoder to measure patient-induced mobility and a second encoder functioning as a force sensor. Unlike previously described devices (34,35), MR_CHIROD is the first ERF-based device that has been demonstrated to function in conjunction with fMRI for brain mapping in chronic stroke patients (33,36). Of note, MR_CHIROD is capable of limiting and controlling a number of factors that affect its function, rendering it particularly useful for home-based training given the low level of expert clinical support in the home environment that can be accompanied by low extrinsic motivation. MR_CHIROD can be re-engineered to improve the cost-to-benefit ratio and therapy effectiveness by providing autonomous and recordable training programs with extrinsic motivation through virtual reality technology.

### Process, training and MRI protocol

As described in a previous study (8), all studies were performed on a state-of-the-art 3-T MR system in order to obtain a high signal-to-noise ratio (SNR). We used a systematic approach to optimize the protocol with respect to SNR by varying the number of echoes, the echo time, the repetition time, the Generalized Autocalibrating Partially Parallel Acquisitions (GRAPPA) acceleration factor, the field of view (FOV), and the number of excitations (NEX). These factors were set in such a manner that the protocol could be completed in 45 min and a 12-channel Siemens Tim coil was used. The functional MRI protocol was as follows: T1-weighted MR images (a high-resolution three-dimensional T1-weighted, MP-RAGE image was obtained for anatomical reference and optimal gray-white matter contrast); fluid attenuation inversion recovery (FLAIR); providing anatomical localization of hyperintense regions and MR images of stroke lesions and Multilevel fMRI (high-resolution GRAPPA EPI sequence for whole-brain BOLD fMRI at optimal spatial resolution for BOLD detection).

Patients completed a single training at home and underwent serial MR evaluation at baseline and after 8 weeks of training. Training at home consisted of squeezing a gel exercise ball with the paretic hand at ~75% of maximum strength for 1 h/day, 3 days/week. For each patient, reference (100%) was own maximum force, defined as the force at which subjects were able to completely squeeze the MR_CHIROD [group max force: 128±13 N (n=5, male)]. All the studies were performed on a Siemens Tim Trio (3T) and BOLD fMRI was performed using GRAPPA gradient-echo EPI (TR/TE = 3,000/30 msec, 1.56×1.56×3 mm). A block design paradigm was used for fMRI. During the action period, subjects squeezed the MR_CHIROD and released continuously. A fixation cross was projected during rest. Each volunteer performed the paradigm at 45, 60, and 75% of their maximum grip strength and fully squeezed the device at all levels. The percentage levels compensate for performance confounds.

**Figure 1:**
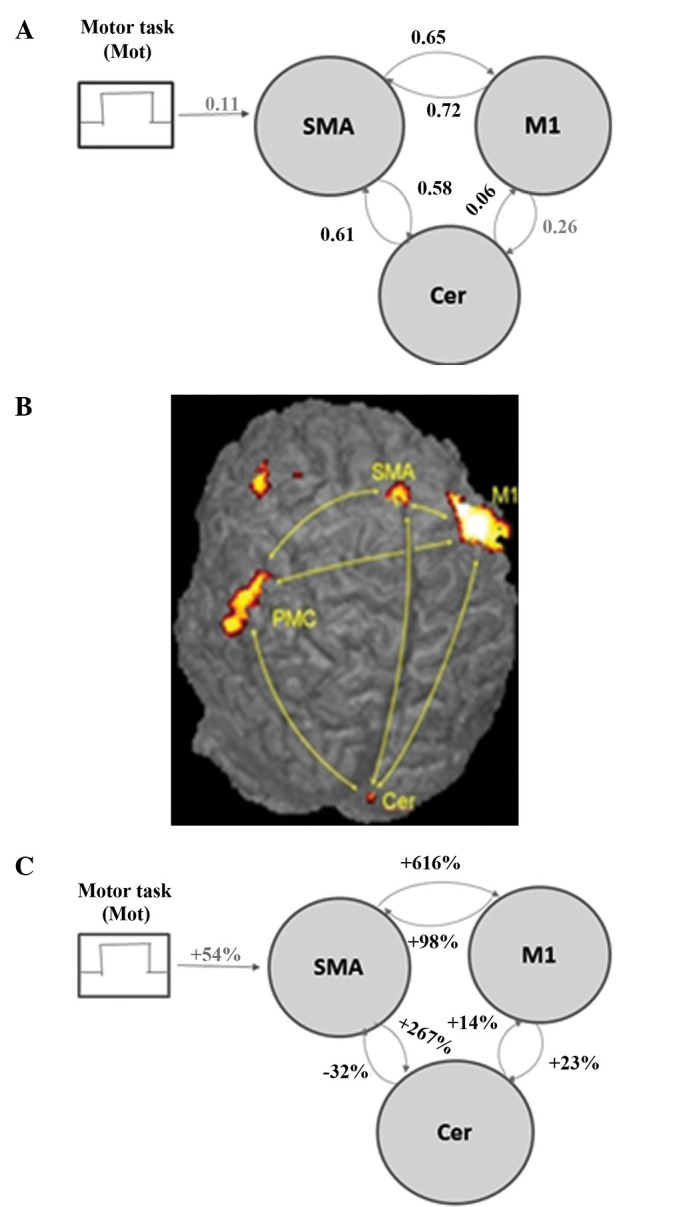
Alterations in connectivity after training relative to baseline in the patients and healthy subjects. (A) The DCM used in this study and DCM connectivity (in Hz) for healthy volunteers. The model for intrinsic connections has links between the primary motor area (M1), supplementary motor area (SMA), and cerebellum (Ce). Possible connections between these areas were allowed to account for plasticity changes in the stroke group. (B) The functional magnetic resonance imaging (fMRI) signals during the motor task superimposed on the brain. (C) The percentage change of connectivity strengths between the brain areas of stroke patients after training (relative to baseline).

The DCM model was constructed for the connectivity analysis using brain regions that were activated in all subjects (Fig. 1A) and comprised three regions: M1, SMA, and Ce. Volumes of interest were defined in these regions using a sphere centered at the maximum activation from the second-level analysis and with a radius of 2 voxels. Possible connections between the brain areas were permitted to account for plasticity changes in the stroke patients. Mot connected to the SMA, which is the only region in the model responsible for motor planning. Connectivity strengths and posterior probabilities were calculated using the DCM utility in SPM5.

### Statistical analysis

Statistical analysis was performed with ANOVA with Least Significance Difference adjustment for post-hoc comparisons, Mixed Model Procedure with Restricted Maximum Likelihood estimation, SPSS version 12.

## Results

The results suggest that a dysfunctional connectivity between SMA and Ce and/or M1 underlies hand motor disability after stroke. We suggest that assessing effective connectivity by means of fMRI and dynamic causal modeling might be used for the evaluation of training-promoting recovery of function and neuroplasticity after stroke.

Table I summarizes the findings of the study. More specifically, the fMRI analysis revealed activations in M1, SMA, premotor cortex and Ce in both stroke patients and controls (Fig. 1B). Greater connection strength translated into a greater absolute value of the parameter shown, and thus a more prominent effect of one area on another (Table I). Connectivity strengths of healthy subjects are shown in Fig. 1A and percentage changes in connectivity strengths after training relative to the baseline are shown in Fig. 1C. The DCM analysis produced the following three noteworthy results: i) in healthy subjects performing a simple motor task, there was minimum effective connectivity from Ce to M1 (Fig. 1A); ii) training significantly increased coupling between M1 and SMA, suggesting an induction of SMA recruitment (Fig. 1C). This possibility has been suggested by earlier fMRI studies in healthy subjects (37). iii) SMA-Ce coupling and Ce-M1 coupling were induced by training (Fig. 1C).

## Discussion

Results of this study indicate that fMRI is a promising molecular imaging procedure and show connectivity alterations in motor-related areas suggesting functional reorganization of motor systems in stroke (8). Of note, enhancement of SMA activity through training has been suggested as a potential means for ameliorating M1 dysfunction after stroke. These results emphasize the importance of the role, of SMA not only for the preparation and execution of intended movements, but also for suppressing movements that are represented in the motor system but are not to be performed. These results also demonstrate that connectivity alterations between motor areas may help balance a functionally abnormal M1 in chronic stroke patients. In a previous study, Ce hyperactivity was also documented in Parkinson’s disease patients, where it was suggested to represent a compensatory mechanism for defective basal ganglia (38). In the present study, Ce hyperactivity reflects efforts by stroke patients to improve motor balance and function. Our results confirm data of a previous study (6). Moreover, in this study, data suggest that a dysfunction between ipsilesional and contralesional M1, and between ipsilesional SMA and contralesional M1 underlies hand motor disability following stroke. Assessing effective connectivity by means of fMRI and dynamic causal modeling might be used in the future for the evaluation of interventions promoting recovery of function.

The present results confirm and extend previous findings in stroke rehabilitation and plasticity. A meta-analysis focusing on 10 studies of robot-assisted therapy on motor and functional recovery in 218 stroke patients showed a significant effect on motor recovery in the upper paretic limb but no significant effect on functionality (39). In another similar report, the authors found no significant improvement in daily activities, although motor function and arm motor strength improved (40). A more recent meta-analysis of 11 eligible studies that included 328 patients showed significant improvements in motor function and strength of the paretic arm with electromechanical and robot-assisted arm training, but there were no improvements in activities of daily living. Robot-assisted therapy has been shown to benefit patients during neurological recovery (21,25,29,32). Specifically, individuals who received robotic therapy exhibited improved gain-in-motor coordination and muscle strength of the exercised shoulder and elbow relative to control subjects (32). Furthermore, Volpe *et al*(25) reported that these improvements were sustained over a 3-year period following inpatient discharge from the hospital.

Our results show the importance of exercise and training after stroke, potentially crucial for a rapid recovery. Recent studies have shown that individuals with stroke, given the opportunity to exercise after stroke, maintain their functional status after the initial rehabilitation and improve function (41,42). In this study, training suggests reorganization in M1, SMA, premotor cortex and Ce, which has been documented in other studies. Reorganization of brain networks has already been explored in humans (43), non-human primates (44) and rats (45). Of note, despite the disrupted motor patterns following stroke, motor system reorganization has been demonstrated in stroke patients (46,47), confirming results of the present study. Our results suggest that patients with minor corticospinal system damage show plasticity in the process of recovery. In a previous study, non-invasive transcranial magnetic stimulation have been used successfully for the activation of SMA, resulting in M1 improvement (48). Since stroke recovery may vary among different cultures, future investigations are required on specific training approaches that should be matched to the individual case characteristics.

In recent years, there has been an explosive research trend to rehabilitative evidence, which formulated a large platform of new technologies (e.g., robotics) and systems for stroke recovery. Virtual reality can engage patients, increase their attention during the task, and improve motivation, thus increasing the effectiveness of rehabilitation. However, more investigation is required for the development of a united code applicable to all settings that potentially lead to the best possible outcomes for stroke survivors.

In conclusion, we suggest that assessing changes in connectivity by means of fMRI and MR_CHIROD might be used in the future to demonstrate the neural network plasticity that underlies functional recovery in chronic stroke patients. Our findings suggest that rehabilitative exercise training might induce functional connectivity alterations after training in both stroke and healthy subjects. Thus, we purport that fMRI as a molecular imaging biomarker of functional reorganization of motor systems in stroke is a clinically relevant molecular medicine approach and it may allow caregivers to select the most appropriate rehabilitation approach for each patient and to fine-tune this approach based on brain maps obtained before and after a short trial of therapy. This is a new concept of personalized molecular medicine combining motor fMRI with a novel MR-compatible hand-induced robotic device training in chronic stroke that can be applied in other motor pathologies.

## Figures and Tables

**Table I:** Connectivity strengths in chronic stroke patients for the selected intrinsic model.

Pathway	Baseline^a^	After training^a^	% Difference from baseline^b^	P-value
M1→SMA	0.50±0.05	49.49±0.07	+98^c^	<0.001
SMA→M1	0.37±0.07	2.65±0.05	+616^c^	<0.001
SMA→Ce	0.32±0.06	0.17±0.08	+267^c^	<0.05
Ce→SMA	0.41±0.04	0.29±0.06	−32	<0.05
Ce→M1	0.35±0.03	0.40±0.05	14	NS
M1→Ce	0.39±0.06	0.48±0.07	23	NS

a Values are means ± SD in Hz.

b Values are the percentage difference between baseline and after training.

c Statistical significance. Statistical analysis was performed as described in Materials and methods.

SMA, supplementary motor area; M1, primary motor cortex; PMC, premotor cortex; Cer, cerebellum; and NS, not significant.
